# To Fake or Not to Fake: Antecedents to Interview Faking, Warning Instructions, and Its Impact on Applicant Reactions

**DOI:** 10.3389/fpsyg.2016.01771

**Published:** 2016-11-15

**Authors:** Stephanie J. Law, Joshua Bourdage, Thomas A. O’Neill

**Affiliations:** Department of Psychology, University of Calgary, CalgaryAB, Canada

**Keywords:** interview faking behavior, impression management, warning instructions, Honesty-Humility

## Abstract

In the present study, we examined the antecedents and processes that impact job interviewees’ decisions to engage in deceptive impression management (i.e., interview faking). Willingness and capacity to engage in faking were found to be the processes underlying the decision to use deceptive impression management in the interview. We also examined a personality antecedent to this behavior, Honesty-Humility, which was negatively related to the use of deceptive impression management through increased willingness to engage in these behaviors. We also tested a possible intervention to reduce IM. In particular, we found that warnings against faking – specifically, an identification warning - reduced both the perceived capacity to engage in interview faking, and subsequent use of several faking behaviors. Moreover, this warning reduced faking without adversely impacting applicant reactions.

## Introduction

Within organizations, personnel selection is a critical human resource function. The aims of selection are to increase the probability of hiring the best candidates (those with the skills to perform the job well) while decreasing the probability of hiring undesirable candidates (those with low job fit with the organization and job). The American Management Association (2008) (2008) estimated that the cost of making a bad hiring decision is 1.4 times an employee’s annual salary, which rises as jobs increase in complexity. Although many different methods for selecting employees exist and have demonstrated predictive validity – such as general mental ability testing, personality testing, and work samples (Schmidt and Hunter, 1998) – interviews have been adopted nearly universally in making hiring decisions (Huffcutt and Culbertson, 2011). Accordingly, it is critical to ensure that the information gathered from the interview is accurate.

One potential obstacle to the success of the interview is impression management (IM). IM tactics are specific strategies used by interviewees to manipulate the image they project to others. For instance, applicants might exaggerate or completely fabricate their job qualifications, flatter or compliment an interviewer or organization, or distance themselves from or lie about past negative events in their job history (Levashina and Campion, 2007). Research indicates that there is substantial variability across individuals in the use of IM, and that job applicants who employ IM tactics in the job interview tend to be evaluated more favorably (Stevens and Kristof, 1995; Ellis et al., 2002; Higgins et al., 2003; Barrick et al., 2009). This is a critical issue because IM usage may introduce systematic inaccuracy into the hiring decision process, reducing the predictive validity of the interview (Posthuma et al., 2002).

Of particular concern is explicitly deceptive IM, which is often described as *interview faking* (in the present paper, we use the two terms interchangeably). Levashina and Campion (2007, p. 1639) define interview faking as “conscious distortions of answers to the interview questions in order to obtain a better score on the interview and/or otherwise create favorable perceptions.” This is different from honest IM, in which applicants use IM to better articulate the skills, knowledge, and abilities that they *do* possess. Levashina and Campion (2007) found that over 90% of applicants tend to report using at least some interview faking, although there is variability across individuals. Importantly, studies utilizing signal detection theory indicate that interviewers are not effective at detecting interview faking (Roulin et al., 2014). Furthermore, although increasing the structure of an interview is often viewed as an avenue to increase its validity (Campion et al., 1997), IM has still been found to impact interviewer evaluations in structured interviews (Ellis et al., 2002; Tsai et al., 2005)^1^, as certain elements of the structured interview may create additional opportunities to use faking behaviors (Levashina and Campion, 2006). In short, interview faking happens in both structured and unstructured interviews, is poorly identified in many cases, and has the potential to impact interview validity.

Given this, the present study aims to enhance our understanding of interview faking in a number of ways. Guided by theory on the factors that influence the decision to engage in interview faking (Levashina and Campion, 2007) we investigate (a) which individuals are more likely to engage in faking behavior, (b) a potential intervention for reducing faking behavior in interviews, (c) reactions to this intervention, and (d) mediating mechanisms explaining how these antecedents may impact faking behavior. Specifically, we investigate the relationship between the trait of Honesty-Humility (Lee and Ashton, 2004) and interview faking, and the proposition that this trait operates by influencing one’s willingness to engage in deceptive IM. Second, drawing inspiration from the personality faking literature (Dwight and Donovan, 2003; McFarland, 2003; Converse et al., 2008), we test the utility of using warnings for reducing interview faking, as well as how applicants react to these warnings. We propose that warnings operate through influencing an applicant’s perceived capacity to use IM, as well as their willingness to use such behavior. We believe that the results of this study will lead to important insights about when interview faking is more or less likely to occur, who is more likely to use this behavior, and why.

### Research on Interview Faking

Research on deceptive IM has been growing steadily over the past decade. One prominent advancement sparking research in this area was the development of a taxonomy and a measurement scale through which to understand interview faking. More specifically, over the course of several studies, Levashina and Campion (2007) developed a measure of a four-factor model of deceptive IM use in the interview. These four factors and their subsequent behaviors are described in **Table 1**, and include (a) slight image creation, which refers to minor attempts at faking to create an image of a good candidate (e.g., overstating job experiences), (b) extensive image creation, which refers to more extreme faking behaviors (e.g., inventing job experiences) (c) image protection, which refers to defensive tactics (e.g., omitting bad past experiences) and (d) deceptive ingratiation, which refers to tactics aimed at increasing likability (e.g., insincerely complimenting the interviewer). This taxonomy was used in this current study.

**Table 1:** Taxonomy of Faking Behaviors (proposed by Levashina and Campion (2007).

Dimensions of faking	Behaviors
Slight image creation	– Embellishing
	– Tailoring
	– Fit enhancing
Extensive image creation	– Constructing
	– Inventing
	– Borrowing
Image protection	– Omitting
	– Masking
	– Distancing
Ingratiation	– Opinion conforming
	– Interviewer or organization enhancing

Interview faking has been found to inflate interviewer evaluations of the candidate (Levashina and Campion, 2007; Swider et al., 2011; Roulin et al., 2014). As not everyone engages in interview faking to the same extent (Levashina and Campion, 2007), this is problematic insofar as some individuals may receive higher interview ratings than warranted, thus jeopardizing the validity of the interview (Posthuma et al., 2002). Troublingly, recent research has found that deceptive IM may go undetected by interviewers. Roulin et al. (2015) investigated IM usage in actual employment interviews, and found little convergence between an interviewer’s perceptions of an interviewee’s use of IM and an interviewee’s self-reported use of IM. This is consistent with low correlations between self-reported IM and observer rated IM reported by Stevens and Kristof (1995). Roulin et al. (2015) also found that interviewers were not very successful at detecting IM overall (ranging from 13 to 23% of IM tactics correctly detected).

Taken together, recent research conducted on IM use suggests that many applicants are able and willing to use deceptive IM in interviews, and that such behavior may impact interview performance, as interviewers struggle to accurately detect such behavior. However, a number of questions remain unanswered regarding interview faking, including the processes underlying, the people most likely to engage in it, and potential ways to reduce it.

#### Processes Underlying Interview Faking Behavior

In order to understand interview faking, its potential antecedents, and the ways in which we can reduce such behavior, it is important to understand the theoretical mechanisms that underlie the decision to use such behavior. Although there are several models of faking (Snell et al., 1999; Goffin and Boyd, 2009; Ellingson and McFarland, 2011), our investigation was guided by the interview-specific model developed by Levashina and Campion (2006). This model proposes that the likelihood of interview faking is determined by the applicant’s assessment of three-factors: (a) *capacity* – one’s perceived ability to engage in response distortion; (b) *willingness* – one’s motivation or inclination to engage in response distortion, and (c) *opportunity* – situational variables that influence response distortion. These mechanisms can guide the search for antecedents of interview faking, as well as ways to reduce interview faking. For instance, a recruitment-focused interview is proposed to increase one’s perceived opportunity to fake, whereas general mental ability is proposed to increase the perceived capacity to fake (Levashina and Campion, 2007). Levashina and Campion’s model is particularly important given that lack of theory has been noted as a key issue in the IM literature (Bolino et al., 2008). Moreover, understanding the theoretical mechanisms underlying faking will shed light on who is more likely to use such behavior and, in turn, how to reduce it.

We begin by formally testing whether these theoretical mechanisms are correlated with deceptive IM usage, as this has not been directly tested to date. While we test willingness and capacity, we did not include opportunity (the third mechanism) as this tends to be a function of the situational context such as number or type of questions, which is something that was held constant across participants in our experimental design.

*Hypothesis 1:* Willingness will be positively related to the usage of deceptive IM tactics.
*Hypothesis 2:* Capacity will be positively related to the usage of deceptive IM tactics.

#### Personality: Honesty-Humility

Personality has been found to be particularly important in understanding IM in the interview and the workplace (Kristof-Brown et al., 2002; Higgins and Judge, 2004; Van Iddekinge et al., 2007). Within the study of IM and faking specifically, individual difference variables can potentially tell us about the nature of such behavior, and who is most likely to use these behaviors. For instance, if faking is the tool of particularly deceptive or lazy individuals, this is more of a concern than if applicants all fake to a similar extent, or if it faking is engaged in merely by highly motivated individuals. Within Levashina and Campion’s (2006) framework, personality tends to operate through impacting either an individual’s perceived willingness or capacity to fake. Although several personality variables are proposed to be associated with interview faking, past research in the realm of workplace IM has found that one personality variable in particular seems to be able to most consistently explain a variety of diverse IM behaviors (Wiltshire et al., 2014; Bourdage et al., 2015). This is the personality variable of Honesty-Humility from the HEXACO model of personality (Lee and Ashton, 2004). We chose to focus on Honesty-Humility due to the fact that it (a) has consistently emerged as the most important personality predictor of IM in the workplace, (b) is most closely theoretically aligned with interview faking, and (c) has strong practical implications for the impact of faking.

Individuals high in Honesty-Humility tend to be more sincere, fair-minded, and humble, whereas those low in Honesty-Humility are more manipulative, self-serving, and believe they are superior to others (Lee and Ashton, 2004). Moreover, Honesty-Humility has been found to be the underlying factor of the “Dark Triad” personality variables (Lee and Ashton, 2005): Machiavellianism (*r* = -0.57), Narcissism (*r* = -0.53), and Psychopathy (*r* = -0.72). Furthermore past research has demonstrated that those low in Honesty-Humility tend to be more manipulative and willing to take advantage of other individuals. For example, Hilbig and Zettler (2009) found that those low in Honesty-Humility opted for more selfish decisions in economic games to benefit themselves, and only used more fair allocations when there was the chance to be punished by the other party. Specific to the study of IM, Honesty-Humility has been shown to be the most robust predictor of a wide variety of workplace IM tactics (e.g., Bourdage et al., 2012, 2015), whereas other traits tend to be less consistent in their relations with IM behavior.

It seems plausible that this manipulative, self-serving behavior would generalize to the interview context. Those low on Honesty-Humility may see interview faking as having instrumental value in the interview, and perceive that applicants are expected to portray themselves in a positive light. Consistent with trait activation theory (Tett and Burnett, 2003), the interview may provide a context that activates expression of the manipulative nature of those low in Honesty-Humility.

Integrating Levashina and Campion’s (2006) theory, one might expect that the reason *why* Honesty-Humility relates to use of faking is that individuals low in Honesty-Humility have an increased *willingness* to use deceptive IM. For example, those low in Honesty-Humility have been found to increase willingness to engage in unethical business practices (Lee et al., 2008). Furthermore, those who are high on Machiavellianism (which has a moderate association with low Honesty-Humility; Lee et al., 2013) have been found to report being more willing to engage in dishonest behavior in the interview (Fletcher, 1990) and to view IM use in the interview as fair (Lopes and Fletcher, 2004). As such, we proposed a mediation model, where personality relates to IM through willingness.

*Hypothesis 3:* There will be a negative relation between Honesty-Humility and deceptive IM, such that individuals lower on this trait will be more likely to engage in deceptive IM.
*Hypothesis 4:* Willingness to fake will mediate the relationship between Honesty-Humility and deceptive IM tactics.

#### Effect of Warning Instructions on Deceptive IM

As was noted above, one of the challenges inherent in interview faking is that interviewers do not seem to be accurate at detecting it (Roulin et al., 2014). As such, an alternate possibility is to find methods that can be used to deter interviewees from using IM in the first place. A parallel research area that has encountered this same issue (i.e., difficulty in detecting faking) is the personality literature. Measures that have been proposed to aid in detection of faking on personality tests – such as social desirability scales – have generally been found to have a host of problems (De Vries et al., 2014), whereas other researchers have sought to find methods of deterring individuals from faking. Perhaps one of the most promising avenues to date has been the use of *warnings*, with research finding that warnings against faking can decrease the motivation of individuals to distort their responses on personality tests (Goffin and Woods, 1995; Dwight and Donovan, 2003; McFarland, 2003).

As such, borrowing from the personality testing literature, we believe that one promising intervention to reduce *interview* faking behavior may be the use of such warnings. Consistent with the theoretical model of interview faking (Levashina and Campion, 2006), warnings should provide a successful avenue to reduce faking, as they may reduce an applicant’s perceived willingness and capacity to engage in interview faking. Importantly, very few interventions to reduce interview faking have been tested to date, and the application of warnings to reduce interview faking is novel. We test whether warnings will reduce interview faking, and the efficacy of several types of warnings. Given that warnings have not been widely used in the interview literature, we draw on the research on personality warnings below.

Of the many different types of warnings, Dwight and Donovan (2003) focused on three: identification, consequence, and combination. Identification involved informing individuals that dishonest responses can be detected. In contrast, consequence involved informing individuals what negative consequences would occur if they engaged in faking behavior. A combination warning included both identification and consequence elements. An example of a combination warning would be that individuals are told that those who engage in faking will be identified, and disqualified as a candidate to be “hired.” Dwight and Donovan meta-analyzed average effect sizes from several studies involving the three types of warnings, and found that there was a mean score difference between the unwarned group and the warned group (*d* = 0.23). Specifically, the warned group had a significantly lower mean score than the unwarned group, suggesting that the warning instructions reduced the score inflation in the warned group.

Beyond these extrinsic considerations around being identified and experiencing negative consequences, there may be a more internal approach that warnings can take. Researchers have suggested that values and morals largely influence one’s beliefs toward faking, and subsequent engagement in faking behavior (McFarland and Ryan, 2000; Goffin and Boyd, 2009). Despite this, there has yet to be warning instructions incorporating morality (Goffin and Boyd, 2009). We believe that moral warnings may prove efficacious in reducing interview faking.

More specifically, a moral warning involves appealing to the ethical values of an individual by highlighting the moral norm around faking. Research demonstrates that in order for people to engage in unethical behavior, they often engage in a socio-cognitive mechanism called moral disengagement (Bandura, 1999; Ogunfowora et al., 2013). In other words, individuals use a number of mechanisms to disengage their moral standards so as to avoid feeling guilty about unethical behavior. These behaviors might include justifying the action or minimizing the wrongness of the action (Bandura et al., 1996). In an interview context, applicants might tell themselves that “everyone fakes in interviews” (something which is not true). Therefore, by using a moral warning, individuals may be less able to morally disengage, and ultimately less willing to engage in interview faking. Furthermore, according to the theory of planned behavior (Ajzen, 1991), an individual’s intentions and subsequent behavior are influenced by three factors: (1) the social norms regarding that behavior, (2) his/her attitude toward the behavior, and (3) his/her perceived amount of control to engage in that behavior. As the moral warning is theorized to make ethical norms more salient, individuals should be less likely to engage in dishonest behavior. By highlighting the wrongness around faking, a moral warning should make individuals less willing to engage in faking.

In the present study we focused on three types of warnings and compared these to an unwarned (control) condition: (a) identification warning, (b) moral warning, and (c) combination warning. Although we believe all three types of warnings will reduce faking, due to a paucity of research in this area, we do not have enough information to propose which will have the strongest effect on faking reduction. As such, we merely hypothesize that each type of warning will reduce faking compared to the unwarned condition.

In addition to a main effect on faking, we also proposed that the reduction in faking behavior would operate through the two interview faking processes of capacity and willingness. Specifically, warnings should reduce applicant’s perceived capacity and willingness to engage in deceptive IM. For instance, an identification warning could reduce an individual’s belief that they have the capacity to successfully fake, whereas a moral warning could reduce an individual’s willingness to fake.

*Hypothesis 5:* Those who received any of the three warning instructions will have lower mean levels of deceptive IM tactic usage than those who did not receive warning instructions.
*Hypothesis 6:* Those who received warning instructions will have reduced (a) capacity and (b) willingness to fake.

#### Applicant Reactions to Warnings

A supplementary focus of this study was to examine applicant reactions to warnings. As interviews often have the dual function to both select and recruit candidates (Chapman and Zweig, 2005), organizations may be cautious in using warnings if they have detrimental effects on applicant reactions. In the present study, we focused on two of the dimensions of applicant reactions from McCarthy et al. (2009) that we believed could be influenced by warnings: (a) performance anxiety, which involves being anxious over the outcome of the test or situation, and (b) procedural justice, which involves the perception of fairness in regards to the test or procedure.

##### Performance Anxiety

Performance anxiety has been studied extensively in evaluation and appraisal contexts. Specifically, a negative relationship between performance anxiety and test performance was found (Hausknecht et al., 2004). This may be problematic, as candidates who experience high levels of performance anxiety may be cognitively overloaded because they are focused on how their answers will influence the outcome. Moreover, interview related anxiety has been found to remain stable over the course of the interview (Young et al., 2004). This prolonged anxiety results in the applicant being less able to process performance-related information for the entire duration of the interview (Barlow, 2002). This could result in well-qualified candidates being unable to demonstrate their true performance level in the interview and being screened out of the process early, resulting in lower predictive validity of the interview. This is supported by the findings of McCarthy and Goffin (2004), as they found that interview anxiety negatively predicted interview performance.

Converse et al. (2008) investigated the effects of warnings on test taking anxiety, and found that applicants in the negatively warned group reported higher test taking anxiety than those in the positively warned and unwarned groups. This makes intuitive sense, as negatively framed warnings involve emphasizing the loss of a desired opportunity or reward if they are being dishonest (i.e., being disqualified from obtaining a monetary reward). In contrast, positively framed warnings involve emphasizing the reward the individual will obtain should they be honest. The authors reasoned that because the negatively framed warning focuses on the negative consequences that will result from response distortion, individuals might be more anxious.

*Hypothesis 7:* Those who received the warning instructions will experience more performance anxiety.

##### Procedural Justice

Procedural justice refers to whether the actual format and content (interview) was perceived as fair. Candidates who experience injustice during the interview process may not recommend the company to other potential applicants (Kohn and Dipboye, 1998). Therefore, it is important to consider how warnings may impact procedural justice.

The specific manner in which interview warnings could impact procedural justice perceptions is unclear. On the one hand, because warning instructions may involve informing the applicant that he or she may be identified and disqualified if dishonesty is detected, this may cause applicants to view the process as less fair, as they feel that the interviewer is distrusting them and lead to lowered perceptions of procedural justice. In contrast, warnings may elicit elevated perceptions of procedural justice, as individuals may perceive warnings as an intervention that can allow for everyone to receive fair and equal consideration in the interview. McFarland (2003) investigated applicant reactions to the combination warning in personality testing. There was no significant difference, however, found between the warned and unwarned group in perceptions of justice. Given the conceptual possibility of a positive or negative relationship, paired with the empirical demonstration of a null relationship in the personality warning literature, we posited the following research question:

*Research Question 1*: How will the warning instructions affect procedural justice perceptions?

## Materials and Methods

### Participants

Participants were 173 undergraduate students enrolled in psychology courses at a university in Western Canada^2^. The age of the participants ranged from 17 to 53, with a mean age of 21.6 years (*SD* = 4.35). The sample was predominately female (75.5%) and 77% of participants had experienced at least 3–4 interviews prior to this study.

### Procedure

Each interview was conducted by one of two interviewers. Due to logistical considerations, we could not have all interviews conducted by a single interviewer, and so split the work between two. Interviewers were recruited and selected from the business program at the university, and were both female and had similar appearance, ethnicity, and mannerisms (e.g., tendency to smile). Both of the interviewers also had prior interviewing experience (1–2 years). Interviewers also wore similar professional clothing to each interview. The interviewers underwent three rounds of practice interviews, in which they were both familiarized with the warning instructions and interview script. A 20 minute semi-structured scripted interview was used in order to keep the number of opportunities available for the applicant to use deceptive IM tactics consistent. Specifically, the interview consisted of both unstructured (i.e., “what would you consider a weakness, or an area that you would like to improve on”), and structured behavioral questions (i.e., “tell me a time when a coworker asked you to set aside your own work to help him or her out. What did you do? Why did you do that? What was the result?”), to better mirror real employment interviews. Jokes were also scripted into the interview for interviewees to have an opportunity to decide whether or not to use ingratiation (i.e., “I don’t really ask people where they see themselves in 5 years anymore. The last time I asked that, someone told me ‘celebrating the 5th anniversary of you asking me this question”).

Interview training was also used in order to standardize the way interviews were conducted. Each interviewer conducted three interviews with graduate students acting as the interviewee. The interviewers discussed any point of the interview that they had dissimilarity in conducting, and agreed upon similar tonal and verbal delivery of each element of the interview. After six interviews, the interviewers had reasonably similar delivery of the scripted interview.

Participants were informed in advance that their participation would involve a job interview. Once they arrived, they were given a job description for an insurance sales associate. They were also given role-play instructions, which asked them to imagine themselves as applicants applying for a position (Heggestad et al., 2006), and informed that the top three candidates determined by their interview performance would receive a cash prize of $50. These instructions are used in tandem to create a realistic and motivated situation, and have been used by many researchers in both interview and personality test taking literatures (McFarland and Ryan, 2000; Mueller-Hanson et al., 2003; Converse et al., 2008). The participants were then given 10 minutes to prepare with the job description, and then randomly assigned to one of the four conditions: (1) unwarned condition, (2) identification warning condition, (3) moral warning condition, and the (4) combination warning condition (see Appendix A). After the interview, the participants were led to a separate room, and completed a survey assessing their use of deceptive IM tactics, demographics, Honesty-Humility, and applicant reactions. In order to ensure that the participants felt comfortable reporting honestly, the participants were told explicitly that the interviewers would not see their responses on the survey, and that their survey answers would not influence their performance ratings in any way.

### Materials

#### Deceptive IM Tactics

Deceptive IM tactics were collected through self-report on the Interview Faking Behavior Scale (IFB; Levashina and Campion, 2007). The four facets of IFB were measured: (a) slight image creation, (b) extensive image creation, (c) ingratiation, and (d) image protection. This measure is currently the most widely used measure of deceptive IM, and was rigorously developed over several studies and involved more than 1,300 interviewees. Cronbach’s alpha was 0.87 for slight image creation, 0.91 for extensive image creation, 0.84 for image protection, and 0.87 for ingratiation.

#### Capacity and Willingness

Five items were created deductively to measure these two elements of response distortion. Three items assessed the capability or confidence one felt in using deceptive IM tactics in the interview (α = 0.78). The three items were “I felt confident in my ability to deceive the interviewer,” “I could have provided inaccurate information about myself without the interviewer knowing it,” and “I could have mislead the interviewer if I wanted to.” Two items assessed the willingness that one had in engaging in deceptive IM tactics (α = 0.65). The two items were “I was more than willing to deceive the interviewer” and “I felt motivated to mislead the interviewer”.

#### Honesty-Humility

The personality dimension of Honesty-Humility was measured with 16 Honesty-Humility items from HEXACO (Lee and Ashton, 2004). Cronbach’s alpha for the overall measure was 0.77. This scale captures four facets that make up Honesty-Humility. Responses were provided on a 5-point Likert scale ranging from 1 (*Strongly Disagree*) to 5 (*Strongly Agree*).

#### Performance Anxiety

Four items as used and adapted by McCarthy et al. (2009) from the Measure of Anxiety in Selection Interviews (MASI; McCarthy and Goffin, 2004) were used to assess performance anxiety (α = 0.79). An example item is: “During the interview, I was worried that my performance score would be lower than that of other applicants.”

#### Procedural Justice

Three out of six items used by McCarthy et al. (2009) from the Selection Procedural Justice Scale (Bauer et al., 2001) were used to examine procedural justice (α = 0.76). An example of an item is: “An applicant who scored well on this interview will be a good employee.” These specific three items were selected due to their relevance to the first selection interview process. The other three items assessed reactions to being invited to a second interview, or other after interview events, such as site visits.

#### Motivation

Four motivational items as adapted by McCarthy et al. (2009) from the 10-item Test Attitude Survey (Arvey et al., 1990) were used to assess participant motivation during the interview (α = 0.78). These items were included as a manipulation check to ensure that participants were motivated to do well in the interview. Responses were assessed on a five point likert scale, ranging from “1” (to no extent) to “5” (to a large extent).

## Results

Means, standard deviations and correlations are displayed in **Table 2**. **Table 3** contains the means and standard deviations of motivation for each respective condition. There were no significant differences between the conditions in motivation [*F*(3) = 2.25, *n.s.*] of the participant. In addition, the overall motivation of interviewees was high (*M* = 4.17 out of 5) indicating that despite being students in an experiment, they took the interview seriously. We also created a dummy coded variable for the two interviewers to test for interviewer effects. There was also no effect of interviewer found on any of the dependent variables, and as such we collapsed across the two interviewers.

**Table 2:** Descriptive statistics and correlations.

Variable	*N*	Mean	*SD*	1	2	3	4	5	6	7	8	9	10
(1) Age	173	21.45	4.11										
(2) Honesty-Humility	173	3.40	0.60	0.07	(0.77)								
(3) Slight image creation	173	1.70	0.53	-0.01	-0.17^∗^	(0.87)							
(4) Image protection	173	1.83	0.55	-0.03	-0.20^∗∗^	0.74^∗∗^	(0.84)						
(5) Extensive image creation	173	1.41	0.48	-0.15	-0.27^∗∗^	0.72^∗∗^	0.61^∗∗^	(0.91)					
(6) Ingratiation	173	1.72	0.60	-0.08	-0.27^∗∗^	0.73^∗∗^	0.64^∗∗^	0.59^∗∗^	(0.87)				
(7) Willingness	173	1.97	0.67	0.00	-0.44^∗∗^	0.37^∗∗^	0.31^∗∗^	0.49^∗∗^	0.28^∗∗^	(0.65)			
(8) Capacity	173	3.15	0.92	-0.02	-0.37^∗∗^	0.17^∗^	0.12	0.25^∗∗^	0.15^∗^	0.55^∗∗^	(0.78)		
(9) Procedural Justice	173	3.03	0.06	-0.02	0.02	-0.03	-0.08	-0.03	0.04	-0.10	0.02	(0.76)	
(10) Performance Anxiety	173	2.46	0.07	-0.17^∗^	-0.15^∗^	0.33^∗∗^	0.42^∗∗^	0.29^∗∗^	0.35^∗∗^	0.04	-0.10	0.01	(0.79)

**N* = 173. *^∗^p* < 0.05, ^∗∗^*p* < 0.01.*

**Table 3:** Means and SD of motivation.

Condition	*N*	Motivation
Unwarned	45	4.06 (0.55)
Identification	41	4.34 (0.58)
Moral	45	4.21 (0.55)
Combination	42	4.06 (0.59)

Hypothesis 1 and 2, which posited that willingness and capacity would be related positively to deceptive IM, were tested through correlational and regression analyses. Seven out of the eight correlations between willingness and capacity and the four deceptive IM tactics were significant, ranging from *r* = 0.12 to 0.49 (see **Table 2**). The sole exception is that the correlation between capacity and image protection (*r* = 0.12, *ns*). On the whole, the average correlation between willingness and deceptive IM is 0.35, and the average correlation between capacity and deceptive IM is 0.17. Furthermore, hierarchical regression analyses were conducted on each deceptive IM tactic (see **Table 4**). When willingness and capacity were simultaneously entered as predictors, willingness remained statistically significant, whereas capacity did not, suggesting willingness provided incremental prediction beyond capacity. Therefore, Hypothesis 1 was supported, whereas Hypothesis 2 was only partially supported.

**Table 4:** Multiple regression of IM, willingness, and capacity.

	Slight image creation				Extensive image creation				Image protection				Ingratiation			
Variable	*b*	*SE*	*t*	*p*	*b*	*SE*	*t*	*p*	*b*	*SE*	*t*	*p*	*b*	*SE*	*t*	*p*
Willingness	0.25	0.05	4.71	0.00^∗∗^	0.29	0.05	6.36	0.00^∗∗^	0.23	0.06	3.99	0.00^∗∗^	0.21	0.06	3.25	0.00^∗∗^
Capacity	-0.03	0.05	-0.54	0.59	-0.02	0.04	-0.39	0.70	-0.04	0.05	-0.81	0.42	-0.01	0.06	-0.03	0.98

**N* = 173. ^∗^*p* < 0.05, ^∗∗^*p* < 0.001.*

**FIGURE 1:**
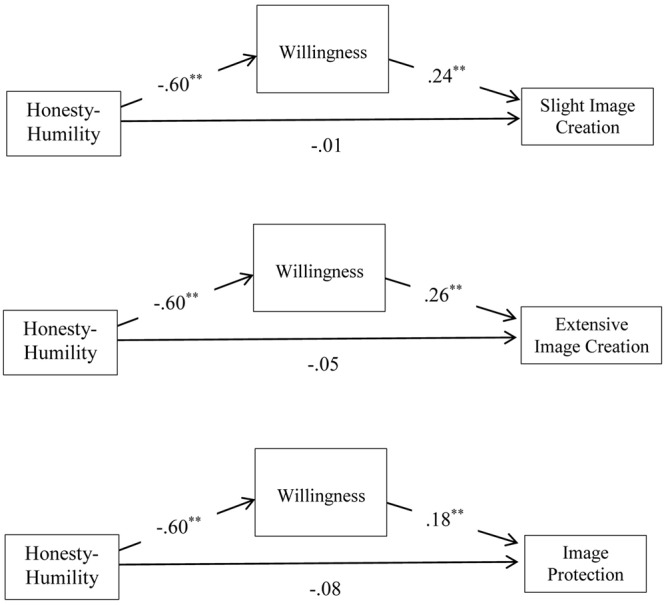
**Indirect effects of Honesty-Humility on slight image creation, extensive image creation, and image protection through willingness.** ^∗∗^*p* < 0.001.

Hypothesis 3 received support, as Honesty-Humility was negatively correlated with the use of all four deceptive IM tactics, including slight image creation (*r* = -0.17, *p* < 0.05), extensive image creation (*r* = -0.27, *p* < 0.001), image protection (*r* = -0.20, *p* < 0.001), and ingratiation (*r* = -0.27, *p* < 0.001). To test the mediation advanced in Hypothesis 4, we used the PROCESS add on to SPSS (Hayes, 2012). A series of sequential mediation models were tested with Honesty-Humility as the predictor, each deceptive IM tactic as a dependent variable, and willingness and capacity as the mediators. There was no direct effect found from Honesty-Humility to each deceptive IM tactic once willingness was included in the model, suggesting the possibility of full mediation. There was, however, a significant effect found from Honesty-Humility to willingness, and willingness to three of the four deceptive IM tactics. More specifically, Honesty-Humility was indirectly related to the use of slight image creation (*a^∗^b =* -0.14, 95% CI [-0.22, -0.07]), extensive image creation (*a^∗^b* = -16, 95% CI [-0.24, -0.09]), and image protection (*a^∗^b* = -0.11, 95% CI [-0.19, -0.04]) through willingness (**Figure 1**). Thus, it appears that those who are high in Honesty-Humility were less likely to engage in deceptive IM tactics through decreased willingness. This supports Hypothesis 4.

To test Hypothesis 5, which stated that those who receive warnings would engage in less deceptive IM, independent sample *t*-tests were conducted. Specifically, each warning condition was compared to the unwarned condition to determine if the mean level of deceptive IM tactic use was significantly different from the warning conditions. Those who received the identification warning engaged in significantly less slight image creation [*t*(84) = 1.77, *p* = 0.040], and extensive image creation [*t*(84) = 2.21, *p* = 0.015] than those who did not receive any warning instructions. However, there was no significant reduction in deceptive IM behavior found between those who received the combination or moral warning instructions and the unwarned condition. Therefore, Hypothesis 5 was partially supported, as only the identification warning reduced deceptive IM usage.

Hypothesis 6 posited that warnings would impact willingness and capacity. The means of willingness, capacity, and each deceptive IM tactic for each condition are displayed in **Table 5**. Two Analyses of Variance (ANOVAs) were conducted with willingness and capacity as the dependent variable, and condition as the independent variable. There was a significant effect of condition on capacity [*F*(3) = 2.86, *p* = 0.04], but no significant effect of condition on willingness [*F*(3) = 1.01, *n.s*]. As such, follow up *t*-tests were used to examine the effect. Hypothesis 6 was partially supported, as the identification warning was found to significantly reduce the capacity to fake [*t*(84) = 1.89, *p* = 0.03] below the unwarned condition. However, there was no significant impact on willingness [*t*(84) = 1.66, *n.s*].

**Table 5:** Means and SD of capacity, willingness, and IM tactics, and *t*-tests for each condition.

						Means (SD)					

**Condition**	***N***	**Capacity**	**Willingness**	**Slight image creation**	***t*-test(df) and *p*^+^**	**Extenive image creation**	***t*-test(df) and *p*^+^**	**Image protection**	***t*-test(df) and *p*^+^**	**Ingratiation**	***t*-test(df) and *p*^+^**
Unwarned	45	3.20 (0.94)	1.99 (0.70)	1.82 (0.62)		1.54 (0.63)		1.91 (0.66)		1.82 (0.70)	
Identification warning	41	2.81 (0.99)	1.87 (0.50)	1.61 (0.49)	1.73 (84), 0.04^∗^	1.30 (0.33)	2.18 (84), 0.02^∗^	1.80 (0.55)	0.84 (84), 0.20	1.62 (0.52)	1.49 (84), 0.07
Moral warning	45	3.37 (0.81)	2.04 (0.73)	1.67 (0.49)	1.27 (88), 0.10	1.42 (0.47)	1.02 (88), 0.16	1.73 (0.45)	1.51 (88), 0.07	1.78 (0.64)	0.28 (88), 0.39
Combination warning	42	3.17 (0.87)	1.94 (0.73)	1.70 (0.53)	0.96 (85), 0.17	1.39 (0.39)	1.32 (85), 0.10	1.88 (0.54)	0.23 (85), 0.41	1.67 (0.48)	1.16 (85), 0.13

*^∗^*p* < 0.05, ^+^one-tailed test.*

To investigate Hypothesis 7 and research question 1, which involved examining the effects of the warning instructions on applicant reactions, two ANOVAs were conducted on procedural justice and performance anxiety. There was no significant effect of condition found on procedural justice [*F*(3) = 1.56, *n.s.*] or performance anxiety [*F*(3) = 0.36, *n.s.*]. **Table 6** contains the mean applicant reactions experienced by the participants in the four warning conditions. Therefore, Hypothesis 7 was not supported.

**Table 6:** Means and SDs of applicant reactions.

		Means (SD) of applicant reactions		
Condition	*N*	Performance anxiety	Motivation	Procedural justice
Unwarned	45	2.49 (0.80)	4.09 (0.57)	2.80 (0.67)
Moral warning	45	2.57 (0.85)	4.19 (0.58)	3.05 (0.73)
Identification warning	41	2.43 (0.95)	4.39 (0.51)	2.98 (0.68)
Combination warning	42	2.36 (0.82)	4.12 (0.63)	3.19 (0.78)

Finally, one interesting set of correlations is between the use of deceptive IM and applicant reactions. It appears that those who used deceptive IM reported significantly higher performance anxiety, with an average correlation of 0.35. On the other hand, deceptive IM was not associated with how individuals perceived procedural justice.

## Discussion

The present study contributes to our understanding of interview faking in a number of ways. First, we tested two components (willingness and capacity) of the model of faking proposed by Levashina and Campion (2006) to better understand the underlying mechanisms of interview faking. Second, we investigated the effect of personality on interview faking, and the processes and mechanisms through which it relates to faking. Third, we investigated the impact of an intervention variable – namely, faking warnings. Fourth, applicant reactions to these warnings were considered as an important outcome for recruitment. Each of these contributions will be discussed further in the following sections.

### Honesty-Humility

Though Honesty-Humility has been found to be related to unethical behavior such as lying, cheating on exams, and counterproductive work behaviors in the workplace (Lee et al., 2005, 2013), there is very little information as to the theoretical mechanisms linking Honesty-Humility to these behaviors. Whereas some warnings against faking seemed to operate through reducing *capacity* to fake, the findings of this study suggest that those who are low in Honesty-Humility tend to have higher *willingness* to engage in deceptive IM tactics. In turn, they tend to engage in more slight, extensive image creation, and image protection behaviors in the interview. Together, this seems to indicate that personality variables and situational interventions likely operate through different mechanisms.

Interestingly, although those low in Honesty-Humility were significantly more confident in their ability to engage in faking behaviors, there was no mediating effect of capacity on actual deceptive IM behavior. Therefore, it appears that Honesty-Humility is a trait related to engagement in dishonest behaviors through motivation, and not the perceived ability to engage in these behaviors. Whereas some may posit that the interview is a strong situation that promotes faking to the extent that “everyone does it,” this research adds to a body of literature indicating that some are more likely than others to do so (e.g., Weiss and Feldman, 2006), and specifically, that these individuals tend to be low in Honesty-Humility. Theoretically, therefore, interview faking is not simply an inevitable part of the interview, as even the less threatening forms of faking, such as slight image creation (exaggerating qualifications or characteristics or stretching the truth) or ingratiation (laughing at the interviewer’s jokes, or flattering the organization or interviewer) are also most likely to be engaged in by low Honesty-Humility applicants. This relation between Honesty-Humility and deceptive IM may be problematic for two reasons. First, research indicates that interviewers are poor at detecting deceptive IM (Roulin et al., 2015), and that interview faking can positively inflate interview performance (e.g., Levashina and Campion, 2007). Second, organizations may be more likely to hire undesirable candidates, as those who are low in Honesty-Humility are more likely to engage in counterproductive workplace behaviors (Lee et al., 2005), make unethical decisions (Lee et al., 2008), and continue to use workplace IM behaviors (Bourdage et al., 2015).

From a practical perspective, these findings indicate that because interviewers are poor at detecting deceptive IM (Roulin et al., 2015), organizations should be identifying and screening out those low in Honesty-Humility during the selection process. Unfortunately, this may be easier said than done. Research to date seems to indicate that detection of Honesty-Humility in a workplace context is generally poor (Bourdage et al., 2015) and that methods such as social desirability measures do not accurately identify low Honesty-Humility individuals (De Vries et al., 2014). On the other hand, research indicates that integrity test scores tend to correlate with Honesty-Humility (Marcus et al., 2007), such that integrity testing could present an avenue for screening for Honesty-Humility – although this requires more substantive research. Therefore, at present, the findings regarding Honesty-Humility may say more about the potential impact of interview faking rather than inform what to do about it. As we note below, this latter question may be partially answered by the findings surrounding warnings.

### Warning Instructions

Perhaps the most impactful and novel finding of the present study is that the identification warning was able to reduce faking among interviewees. More specifically, those who received the identification warning reported engaging in less slight and extensive image creation than those in the unwarned condition. When considering the nature of the various faking behaviors, reducing these two classes of behaviors may be particularly important for interview validity, as they involve exaggerating or even completely fabricating experience, qualifications, and characteristics.

Drawing on the theoretical mechanisms identified by Levashina and Campion (2006), the present study attempted to identify *why* warnings would or would not work. Our findings indicate that only identification warnings reduced the applicant’s perceived *capacity* to fake, and a subsequent reduction in faking behavior. The prominent role of the identification warning in reducing perceived capacity to fake makes particular sense, given that capacity taps into whether the applicant believes they can fool the interviewer without them knowing it. In addition, expectancy theory (Vroom, 1964) may explain why identification warning reduced the capacity to fake, but not willingness. Specifically, individuals may be willing to engage in faking in the interview; however, as the risk of being detected increases, individuals may be less *confident* in their capabilities to engage in IM behaviors. Therefore, though they may want to engage in interview faking, they may feel that they do not have the ability to do so. In essence, the interviewee’s expectancy that an attempt to fake will be successful should decline. This should reduce the chances that an interviewee will choose to engage in faking behaviors. However, we do note that the Cronbach’s alpha of the willingness measure was lower, and may have contributed to the lack of significance in regards to willingness.

However, while the identification warning had some success, we should note that neither the combination warning nor the moral warning significantly reduced IM behaviors. The combination warning contains the notion that there will be a negative consequence if one is identified. Although we believe this should act as a deterrent, in the present study, the warnings were positively framed. As such, consequence warnings focused on positive consequence of being able to be considered as a candidate, should their answers be honest. In the personality literature, Converse et al. (2008) found that positively and negatively framed warnings did not differ in effectiveness at reducing faking. However, a positive warning may have not highlighted that faking will lead to a punishment (instrumentality) strongly enough. Given this, future research should compare the effectiveness of positively versus negatively framed warnings in the interview.

The introduction of the moral warning integrated a relatively new suggestion to manage faking. Specifically, previous research has speculated that moral warnings may be an effective means of reducing faking behavior through making morality salient (Robie et al., 2007; Goffin and Boyd, 2009). Unfortunately, there was no significant reduction found in willingness or capacity, although there was a trend toward significance for slight image creation and image protection (*p <* 0.10). This could be due to the fact that moral warnings emphasize the social norm of honesty, but do not include tangible consequences or risk of being identified as dishonest. Despite reinforcing a negative social norm toward faking in the interview (cf. theory of planned behavior, Ajzen, 1991), it appears that the individual’s own attitude toward that behavior, and their perceived control over that behavior may not have been impacted by the moral warning. Thus, perhaps some individuals still chose to engage in deceptive IM behaviors, even when primed to consider the moral issues involved. Future research could focus on investigating the moral warning further, as it has not been extensively studied as a warning.

### Applicant Reactions

The present study did find that some types of warnings – specifically, identification warnings – can reduce faking. However, a critical component of personnel selection involves balancing the enhancement of predictive validity (i.e., a selection goals) with a consideration of how applicants will react to the process (i.e., a recruitment goals). Given this, one potential concern about warnings was that they could adversely impact applicant reactions. We found that there was no detrimental impact of any of the three warning types on either of the applicant reactions we investigated – performance anxiety and procedural justice. This is important for several reasons. From a procedural justice perspective, research shows that lower perceptions of procedural justice lead to negative applicant reactions (Kohn and Dipboye, 1998). The fact that identification warnings could reduce faking without negatively impacting applicant reactions is a further point in favor of the use of warnings.

On the other hand, there was a concern that warnings might impact performance anxiety. Given that performance anxiety has been found to negatively impact interview performance (McCarthy and Goffin, 2004), this is potentially problematic if interviewee performance is impacted. Although we did not find that warnings impacted anxiety, there was a relationship found between those who used deceptive IM tactics and performance anxiety. In short, those individuals who reported faking also reported experiencing more performance anxiety. Moving forward, this presents a potentially important avenue for future research. It may be that performance anxiety is a cue, or signal to the interviewer that the interviewee is engaging in deceptive IM behaviors. Given that past research shows the difficulty of identifying those engaging in deceptive IM (Roulin et al., 2015), this is a potentially fruitful area for further research.

Practically, organizations may want to consider adding warning instructions to their interview process. As that there are no detrimental effects on applicant reactions, warnings may serve as an effective low cost addition to reducing deceptive IM in the interview.

### Strengths and Limitations

One strength of this study was the experimental design, in that random assignment was used to place participants into four different conditions (i.e., three different warning instructions and a control condition). Though field studies may provide more generalizability, it would not be possible to randomly assign candidates to conditions and infer cause and affect relationships. Second, this study employed the use of a semi-structured scripted interview in order to keep the number of opportunities available to fake consistent, while mimicking a real employment interview. Jokes and unstructured questions (e.g., “what is your greatest strength”) were scripted into the interview to enhance the realism. The base rates of deceptive IM tactics overall found for this study were 1.82 (*SD* = 0.57) for slight image creation, 1.46 (*SD* = 0.50) for extensive image creation, 1.87 (*SD* = 0.57) for image protection, and 1.67 (*SD* = 0.59) for ingratiation. These results are highly comparable to the findings of Levashina and Campion (2007), as the base rates of the four deceptive IM tactics in their undergraduate sample in mock interviewers were 1.85 (*SD* = 0.69) for slight image creation, 1.38 (*SD* = 0.56) for extensive image creation, 1.78 (*SD* = 0.72) for image protection, and 1.90 (*SD* = 0.90) for ingratiation. Third, the interviewers were selected carefully based on their appearances and mannerisms in order to maximize their similarity, and they were thoroughly trained in the interview protocol. Furthermore, statistical tests of interviewer effects on the dependent variables were carried out, and no significant differences were found. In sum, we took several steps to ensure that the interview closely mirrored real interviews, with the added benefit of allowing inference of causal relationships.

One limitation to this study was the undergraduate student sample. We tried to alleviate this concern in several ways. First, we offered a monetary reward for high performance to motivate participants. The use of role-play instructions in conjunction with a monetary award to create similar motivation from the participants is commonly used in the personality testing and interview literatures (McFarland and Ryan, 2000; Mueller-Hanson et al., 2003; Converse et al., 2008). Second, as we noted above, our interviewers were thoroughly trained and had experience interviewing. Students and the interviewers were also encouraged to dress professionally. Moreover, the base rates of IM were similar to other studies (Levashina and Campion, 2007), and thus we believe participants were engaged and challenged, and that the experimental design offered a unique opportunity to investigate the psychological processes related to faking. The processes investigated here, in our view, should not be different in actual job applicants.

Despite the use of a student mock interview sample representing a limitation, it also provides opportunities not present in a field sample. We are studying faking, and therefore, self-reports of this behavior are critical. We do note the potential of common method bias with using self-report to assess Honesty-Humility and deceptive IM usage. With self-ratings of Honesty-Humility, there is the concern that an individual may inflate their scores due to egoistic or social desirability reasons. However, using another source for these ratings may not be a good alternative solution. First, research has found that Honesty-Humility is a trait that observers struggle to accurately assess, especially behaviors related to the low end of the trait (Lee et al., 2010). Furthermore, as we have noted, observers are not particularly adept at recognizing IM in the interview (Stevens and Kristof, 1995; Roulin et al., 2015) or workplace (Bourdage et al., 2015) contexts. Thus, self-reports are used as the primary source of measurement in the interview faking literature, despite the possibility of common method bias (see Tsai et al., 2005; Levashina and Campion, 2007; Bolino et al., 2008; Levashina et al., 2014). Moreover, this necessary reliance on self-reports makes the use of experimental or student samples useful, as it is very unlikely that candidates vying for an actual position would admit to fabricating their responses or even being willing to fake. These two concerns (i.e., honest responding and the necessity to use self-reports) likely explains why student samples in this field of research can be ideal (Kristof-Brown et al., 2002; Peeters and Lievens, 2006; Van Iddekinge et al., 2007). Moreover, meta-analytic findings indicate that the role of IM may be similar between field samples and student samples (Barrick et al., 2009). Nonetheless, future studies should be conducted with applicants in a high stakes interview situation, as this could perhaps impact the effectiveness of certain warnings, such as the consequence warning (being disqualified from a reward is likely very different in valence from missing out on a job). A manipulation check, however, was conducted in this study to ensure that the interview was perceived to be realistic. It was found that participants were highly motivated to perform well on the interview, and that the realism of the interview was high.

As we note above, some of the warning instructions used a positive frame instead of a negative framing, (ex. if you respond honestly, you will be considered for the position” instead of “if you respond dishonestly, you will be disqualified from obtaining the position”), which may have resulted in a more modest estimation of the actual effect warnings may have on reducing IM behavior. Though Converse et al. (2008) did not find significant differences between negative and positive framed warnings in terms of effectiveness, this study was conducted in the personality test faking literature, and may differ when applied to the interview context. Future studies could compare the use of positive and negative framed warnings in the interview context, and whether there is a significant influence on IM behavior and applicant reactions.

## Conclusion

This study examined the antecedents of interview faking behavior, the application of a novel method to reducing IM tactic usage in the interview, and the processes through which personality and warnings impact deceptive IM. Willingness and capacity were found to be the processes underlying the decision to use deceptive IM in the interview. Moreover, Honesty-Humility was related to increased use of slight and extensive image creation, and image protection tactics, and it appears that this occurs through the mechanism of willingness. Finally, identification warnings were found to reduce the mean level of some deceptive IM behaviors (slight and extensive image creation), and to reduce perceived capacity to engage in IM successfully. Future research should further examine the use of warning instructions on IM tactic usage, as it appears to be a promising method to reducing faking behavior in the interview.

## Author Contributions

SL conducted the research, analyzed the data, and wrote up the preliminary document. JB contributed by providing edits on various versions of the document, and advice on the data analysis. TO contributed through providing edits on various versions of the document.

## Conflict of Interest Statement

The authors declare that the research was conducted in the absence of any commercial or financial relationships that could be construed as a potential conflict of interest.

## Footnotes

1 Note, however, that much of the studies on IM and its impact on interview performance have not distinguished between honest and explicitly deceptive IM (i.e., interview faking).

2 We obtained study approval from the CFREB at the University.
