# Supplementary material for: To Fake or Not to Fake: Antecedents to Interview Faking, Warning Instructions, and Its Impact on Applicant Reactions
Source: Front Psychol. 2016 Nov 15;7:1771. doi: 10.3389/fpsyg.2016.01771 (PMC5108801; doi:10.3389/fpsyg.2016.01771)
Supplement: Supplementary file 1 [file Data_Sheet_1.docx]

Appendix A

*Warning instructions for each condition*

| Condition | Warning Instructions |
| --- | --- |
| Moral warning condition | “Before we start, I just want to say that it’s the right thing to do to be honest. Being completely truthful in the interview will make it fair for you and other applicants. So be honest with me.” |
| Identification warning condition | “Before we start, I just want you to know that I will be asking some questions that have been adapted from an assessment designed to measure honesty. A lot of research does show that by analyzing the responses to these questions, you can effectively identify how truthful the responses were. So be honest with me.” |
| Combination warning condition | “Before we start, I just want you to know that I will be asking some questions that have been adapted from an assessment designed to measure honesty. A lot of research does show that by analyzing the responses to these questions, you can effectively identify how truthful the responses were. If the analysis of your responses later show that you were being honest, your interview performance will be considered when I select the top 3 candidates for the $50 dollar prize. So be honest with me.” |
